# Recent Progress in Adipic Acid Synthesis Over Heterogeneous Catalysts

**DOI:** 10.3389/fchem.2020.00185

**Published:** 2020-03-31

**Authors:** Wenjuan Yan, Guangyu Zhang, Jinyao Wang, Mengyuan Liu, Yu Sun, Ziqi Zhou, Wenxiang Zhang, Shuxia Zhang, Xiaoqiang Xu, Jian Shen, Xin Jin

**Affiliations:** ^1^State Key Laboratory of Heavy Oil Processing, Center for Chemical Engineering Experimental Teaching, China University of Petroleum, Qingdao, China; ^2^Oil Production Group#2, Huabei Oil Field Company at PetroChina, Langfang, China; ^3^College of Environment and Resources, Xiangtan University, Xiangtan, China

**Keywords:** nanostructured catalyst, glucose, glucaric acid, adipic acid, cyclohexanone, polyoxometalates

## Abstract

Adipic acid is one of the most important feedstocks for producing resins, nylons, lubricants, plasticizers. Current industrial petrochemical process, producing adipic acid from KA oil, catalyzed by nitric acid, has a serious pollution to the environment, due to the formation of waste nitrous oxide. Hence, developing cleaner methods to produce adipic acid has attracted much attention of both industry and academia. This mini-review article discussed advances on adipic acid synthesis from bio-renewable feedstocks, as well as most recent progress on cleaner technology from fossil fuels over novel catalytic materials. This work on recent advances in green adipic acid production will provide insights and guidance to further study of various other industrial processes for producing nylon precursors.

**Graphical Abstract:**
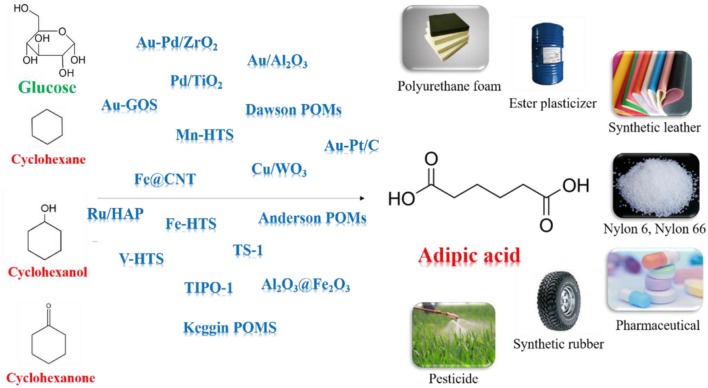
Heterogeneous Catalysts for Adipic Acid Synthesis.

## Introduction

Adipic acid (AA) has immense practical use in industrial for the production of nylon-66, nylon-6, lubricant and plasticizer (Feng et al., 2019; Perkel and Voronina, 2019; Pisk et al., 2019; Yang B. et al., 2019; Yang J. et al., 2019). In current industrial processes, AA is synthesized mainly by oxidation of KA oil using 50–60% nitric acid as oxidant and copper/ammonium metavanadate as the catalyst (Van de Vyver and Roman-Leshkov, 2013; Deng et al., 2016; Rahman et al., 2016). However, this process emits nitrous oxide which can cause ozone depletion, acid rain, and global warming. Furthermore, the applicability of the phase-transfer catalyst in industrial scale is expensive. Obviously, we need to develop more sustainable AA manufacturing process which can avoid the use of toxic reagents and tedious products separation (Dugal et al., 2000; Cheng et al., 2007; Fujitani et al., 2009; Jin et al., 2011; Indulkar et al., 2012; Lu et al., 2012; Vafaeezadeh et al., 2012).

Cyclohexane, cyclohexanol, cyclohexanone can be oxidized to produce AA without formation of any greenhouse gases (Sato et al., 1998; Chatterjee et al., 2018; Luo et al., 2018; Mazzi et al., 2018; Mouheb et al., 2018; Wang et al., 2018). Oxygen, air, hydrogen peroxide (H_2_O_2_) are regarded as clean oxidant since they give water as the only byproduct. It is essential to use separable and reusable inexpensive catalysts for development of sustainable protocols (Baig and Varma, 2012). Various of solid supported catalysts, such as metal oxides, (Hereijgers and Weckhuysen, 2010; Makgwane and Ray, 2014) hollow structure silicates, (Dai et al., 2016; Xia et al., 2018) carbon nanotubes (CNTs), (Machado et al., 2014; Yang et al., 2016) and polyoxometalates (POMs), (Luo et al., 2018) show remarkable performances in AA synthesis, due to the inherent adsorptive properties and tunable acidity.

Some alternative bio-derived AA processes have been extensively reported for synthesizing AA by oxidizing lignocellulosic biomass derived chemicals, e.g., hemicellulose, cellulose, and lignin (Vardon et al., 2015; Han, 2016). Different processes including glucose to glucaric acid process, hydroxymethylfurfural (HMF) to furan dicarboxylic acid (FDCA) process, γ -valerolactone process, lignin and lignin-derived oils process, were reported for AA synthesis from biomass feedstocks (Deng et al., 2016; Gunukula and Anex, 2017; Skoog et al., 2018). In the glucose conversion route, glucaric acid was formed as intermediate by oxidizing the glucose and further undergo hydrogenolysis to form AA (Zhang and Deng, 2015; Zhang and Huber, 2018). This reaction can be achieved in the presence of Au, Pt, and Pd catalysts (Ibert et al., 2002; Merbouh et al., 2002). In the FDCA process, FDCA were formed as intermediates by oxidizing the HMF and was further hydrogeneolyzed to form AA (Gilkey et al., 2018). Noble Pt and Au metals-based catalysts were reported most effective for this reaction (Kong et al., 2018).

Most recent review articles on the synthesis of AA have been listed in this section. Van de Vyver and Roman-Leshkov (2013), Deng et al. (2016), and Rahman et al. (2016) summarized the performances of various catalysts for AA production, with specific focus on metal catalyst design and reaction mechanism. The progress of the metabolic pathways for AA production has been reviewed (Polen et al., 2013; Alonso et al., 2015; Deng et al., 2016; Kruyer and Peralta-Yahya, 2017; Skoog et al., 2018). In 2018, Li et al. (2018) summarized the conversion of cellulose and its derivatives to various organic acids. In this mini review, the glucose and HMF processes will be reviewed systematically. We will particularly focus on the advances of the performances of metallic solid catalysts and POM catalysts for synthesis of AA from both glucose and HMF routes in past 5 years, including mechanistic insights and catalysts stability. The opportunities and challenges in the green process of AA production will be discussed.

## Heterogeneous Metallic Catalyst for Glucsoe and Derivatives Oxidation

**Figure 1:**
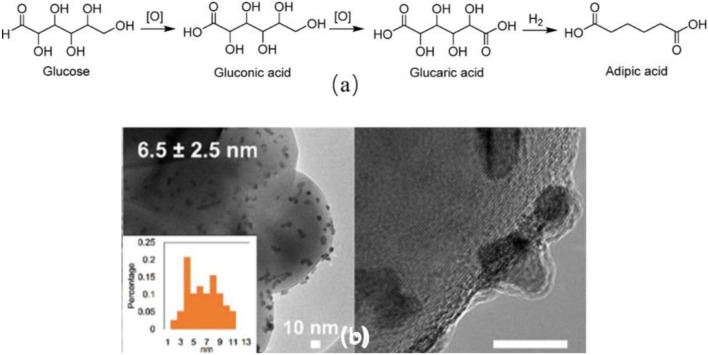
**(A)** Glucose oxidation to produce AA, (Jin et al., 2016). **(B)** TEM images of PtPd/TiO_2_ (Jin et al., 2016).

The transformation of bio-based glucose and its derivatives into AA is green and sustainable. In the first step, the glucose was oxidized to form glucaric acid which was further converted to AA by a catalytic hydrodeoxygenation (HDO) process (Figure 1A). A patent disclosed a yield of 89% of AA in the HDO process over Pt/Rh metallic catalysts in acidic condition using acetic acid and HBr as solvent (Boussie et al., 2014). Lin et al. (2019) reported the deoxydehydration of cellulose-derived D-glucaric acid to AA ester over ReO_x_/ZrO_2_-Pd/C catalysts (Y = 82%). In this part, we summarized the most recent progress of glucaric acid synthesis from glucose.

In the industry, this process can be achieved in the presence of homogeneous catalysts and toxic oxidants under harsh conditions (Smith et al., 2012). The difficulty of separation and the hazardous byproducts hampered the further development of this process. Literatures have widely demonstrated the synthesis of glucaric acid over the supported noble metal catalysts, e.g., Pd, (Jin et al., 2016) Pt, (Bellardita et al., 2016; Shi et al., 2018) and Au (Wojcieszak et al., 2016; Derrien et al., 2017; Solmi et al., 2017) catalysts. Au nanoparticles were immobilized on active carbon (Table 1, #1) (Solmi et al., 2017). After adding Bi additives, AuBi/AC catalyst showed higher glucaric acid yield (Table 1, #2). They claimed that Au particle size affected the ratio between the parallel reactions of gluconic and glucaric acid formation. The reuse study showed a little decline of the activity due to the agglomeration of nanoparticles and the deposition of organic residues (Solmi et al., 2017). Au-Pt and Au-Pd catalysts were supported on various metal oxides (Table 1, #3) (Derrien et al., 2017). The catalytic performance of these catalysts was significantly influenced by the nature of the support. The best glucaric acid yield (44%) was obtained in the presence of ZrO_2_ supported Au-Pt catalyst under base-free conditions (Derrien et al., 2017) CeO_2_ supported Au-Pt catalyst showed the lowest activity. They also noticed that Au-Pd showed higher ability to convert glucose to gluconic acid, but lower ability to further convert gluconic acid to glucaric acid comparing to the Au-Pt catalysts (Derrien et al., 2017). The recycled Au-Pt/ZrO_2_ catalyst was stable in three successive runs, but displayed lower glucaric acid selectivity in the fourth to sixth runs. The TEM images showed the particles' morphology did not change after 24 h reaction. ICP results showed there was no trace of Au or Pt presented in the reaction solution. Hence, they concluded that the activity decline was caused by the multiple handling and washing of the catalyst. The same group synthesized Pt/C catalyst and obtained a yield of 54% of glucarate under alkaline conditions (Table 1, #4) (Derrien et al., 2016). Lee et al. (2016) get a maximum yield of 74% of glucaric acid with Pt/C in aqueous solution with pH of 7.2 using air as oxidant. They found that the selectivity to gluconic acid was higher in acidic conditions due to C-C bond cleavage to short chain carboxylic acids (Table 1, #5) (Lee et al., 2016). The Pt/C catalyst showed good stability in at least five consecutive runs and had no Pt leaching and morphology changing during the reaction. Bimetallic PtPd/TiO_2_ (TOF = 2404 h^−1^) catalyst was synthesized via a simple *in situ* reduction method and displayed much higher activity compared to monometallic catalysts (TOF = 248 h^−1^) due to the existence of PtPd alloy structure as confirmed by the TEM image (Figure 1B, Table 1, #6) (Jin et al., 2016). The PtPd/TiO_2_ catalyst was stable in three consecutive runs with no activity loss, but about 4% Pt and Pd leaching was observed. It is highly possible that the leached metal species may be inactive in this reaction. The same group prepared Pt-Cu/TiO_2_ catalyst using NaBH_4_ as reducing agent and demonstrated a satisfactory activity for glucaric acid (*X* = 92%, *S* = 60%) under base-free conditions (Table 1, #7) (Jin et al., 2015; Shi et al., 2018). They observed strong metal-support interaction between Pt and TiO_2_ support. The stability study showed that the catalyst exhibited same conversion of glucose and marginal change of selectivity to glucaric acid after three runs. This work demonstrated that it is practicable to replace the second noble metal with inexpensive Cu metal for the glucose oxidation process (Shi et al., 2018).

**Table 1:** Heterogeneous metallic catalyst for glucose and derivatives oxidation.

**#**	**Catalyst**	**Reaction conditions**	**Conversion, selectivity**
1	Au/C	Glucose, 60°C, 3 h, 1MPa, O_2_	Y = 24%
2	AuBi/C	Glucose, 60°C, 3 h, 1MPa, O_2_	Y = 31%
3	Au-Pt/ZrO_2_	Glucose, 100°C, 4 h, 4MPa, air	Y = 44%
4	Pt/C	Glucose, 60°C, 24 h, 0.1MPa, air	Y = 54%
5	Pt/C	Glucose, 80°C, 10 h, 1.4MPa, O_2_	X = 99%, S = 74%
6	PtPd/TiO_2_	Glucose, 45°C, 24 h, 0.1MPa, O_2_	X = 100%, *S* = 40.4%
7	PtCu/TiO_2_	Glucose, 90°C, 12 h, 1.5MPa, O_2_	X = 92%, *S* = 60%
8	AuPd/AER	HMF, 100°C, 4 h, 1MPa, O_2_	X = 100%, S = 93.2%,
9	AuPd/CaMgAl	HMF, 100°C, 6 h, 0.5MPa, O_2_	X = 96.1%, S = 89.4%
10	PdNi/Mg(OH)_2_	HMF, 100°C, 10 h, 0.1MPa, air	X = 99%, S = 76%
11	PdCo/Mg(OH)_2_	HMF, 100°C, 10 h, 0.1MPa, air	X = 94%, S = 46%
12	PdCu/Mg(OH)_2_	HMF, 100°C, 10 h, 0.1MPa, air	X = 81%, S = 41%
13	Pt-Ni/AC	HMF, 100°C, 6 h, 0.4MPa, O_2_	X = 100%, S = 43.1%
14	Pt/C	HMF, 110°C, 12 h, 1MPa, O_2_	X = 99%, S = 96%
15	Ru/MnCo_2_O_4_	HMF, 120°C, 10 h, 2.4MPa, air	X = 100%, *S* = 99.1%
16	Ru/HAP	HMF, 120°C, 24 h, 2MPa, air	X = 100%, S = 99.6%

**Figure 2:**
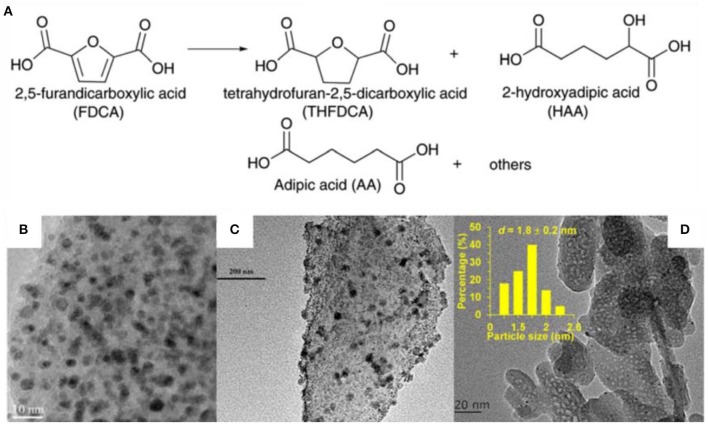
**(A)** HMF to AA, (Lee et al., 2016) TEM images of **(B)** AuPd/CaMgAl, (Gao et al., 2017) **(C)** Pt-Ni/AC, (Shen et al., 2018) **(D)** Ru/HAP (Gao et al., 2018).

HMF is an important platform chemical which can be converted to AA by two steps. HMF was oxidized to form FDCA which undergo deoxygenation to form AA. Wei et al. (2019) reported one-step conversion of FDCA to AA in water over niobic acid-supported Pt catalyst 38% AA yield was obtained at 200°C in 8 h over Pt/Nb_2_O_5_ catalyst which was proved to be stable in at least five repeated runs. The hydrodeoxygenation of FDCA was also conducted in the presence of Pt-MoO_x_/TiO_2_ catalyst with AA yield of 21% at 200°C in 4 h (Asano et al., 2016). The low solubility of FDCA in water may cause the low AA yield. Gilkey et al. (2017) studied the metal-free hydrogenolysis of tetrahydrofuran-2,5-dicarboxylic acid (THFDCA) to produce AA. A 99% THFDCA conversion and 89% yield of AA were obtained at 160°C in 2 h. The literatures about this step was rare, but FDCA synthesis from HMF oxidation has been widely reported as one of the key steps of biomass conversion to AA (Figure 2A) (Zhang et al., 2015, 2018; Zhou et al., 2016; Diamond et al., 2018; Li et al., 2018; Rathod and Jadhav, 2018; Ventura et al., 2018). Au-Pd alloy nanoparticles were immobilized on basic anion-exchange resin and catalyzed the HMF to FDCA reaction with a yield of 93.2% (Table 1, #8) (Antonyraj et al., 2017). The physical mixture of Au and Pd nanoparticles showed only 52% FDCA yield. This confirmed the major role of the AuPd alloy as active species as evidenced by the XPS study (Antonyraj et al., 2017). This catalyst had no metal leaching and activity decreasing after six cycles. Au-Pd alloy was also supported on La-doped CaMgAl layered double hydroxide (LDH) (Gao et al., 2017). TEM images showed that small nanoparticles with 3–4 nm particle size were well-dispersed on the LDH support (Figure 2B) (Gao et al., 2017). A yield more than 99% of FDCA was obtained ascribing to the high surface basicity of the support and the synergy between Au-Pd nanoparticles (Table 1, #9). They also observed that the La_2_O_3_ on the surface of the LDH support can form carboxylic acid products and prevent the deterioration of the LDH support, thus enhance the catalyst stability (Gao et al., 2017). This catalyst maintained good activity after four runs with only 2% decreasing of the yield. No leaching of Au or Pd was detected. However, there were 0.8% of Mg and 0.3% of Ca lost after the reaction.

Ni, Co, and Cu metals were selected to synthesis bimetallic Pd catalysts. Gupta et al. (2017a,b) found that PdNi/Mg(OH)_2_ catalyst displayed higher catalytic performance than Co and Cu based Pd/Mg(OH)_2_ catalyst due to the synergistic cooperation between Pd and Ni species (Table 1, #10–12). This catalyst can be reused for three consecutive reactions without significant activity loss, Pd/Ni metal leaching, or particle size changing. Ni and Pt bimetallic nanoparticles were supported on active carbon by atomic layer deposition method (Table 1, #13) (Shen et al., 2018). The TEM images showed that the metal were uniformly dispersed on the support (Figure 2C). A 97.5% yield of FDCA (TOF= 35.8 h^−1^) was obtained in 15 h reaction. They claimed that the presence of Ni species enhanced the ability of Pt to adsorb and oxidize C = O bond (Shen et al., 2018). The catalysts recycled after four runs showed 86.3% FDCA yield which is lower than the fresh catalysts (97.5%). However, the reasons for the activity loss was not discussed in this work. Pt supported on carbon displayed 96% yield of FDCA in the absence of base (Table 1, #14) (Han et al., 2016). The introduction of N atom brought more medium strength basic sits to the catalyst and thus elevated the catalytic activity (Han et al., 2016). Ru immobilized on MnCo_2_O_4_ was reported highly active (Y = 99.1%) for HMF oxidation under base-free condition (Table 1, #15) (Mishra et al., 2017). The existence of both Lewis and BrØnsted acid sites facilitated the HMF oxidation to FDCA (Mishra et al., 2017). The reusability study showed that there was no significant change in the rate of HMF conversion in at least five successive runs. The TEM images of both fresh and used catalysts indicated that there was no discernible change of the structure. No Ru metal leaching was detected by ICP analysis.

Gao et al. (2018) supported Ru on hydroxyapatite. The TEM image showed a typical rod-shape agglomerates with the mean size of Ru nanoparticles about 1.8 nm (Figure 2D). Hundred percentage of conversion of HMF and 99.6% selectivity to FDCA were obtained in the presence of oxygen and water. The acidic-basic sites on hydroxyapatite support were essential for good catalytic performance (Table 1, #16) (Gao et al., 2018). There was about 10% loss of the FDCA yield after the fifth runs. ICP results revealed there was no leaching of Ru and Ca species from the catalyst. No aggregation of Ru nanoparticles was noticed from the TEM images. The adsorption of impurities and the partial oxidation of Ru nanoparticles were the main reason of the catalyst deactivation.

## Cyclohexane, and Cyclohexanone/Cyclohexanol Oxidation to AA

Cyclohexane and cyclohexanone/cyclohexanol are the most selected chemicals as the model substrates for oxidation reaction to produce AA over various of catalysts, such as metal oxides, carbon nano tubes (CNTs), and TS-1 catalysts (Cavani et al., 2011; Alshammari et al., 2012; Dai et al., 2016; Chen et al., 2017; Nale et al., 2017).

### Cyclohexane Oxidation to AA

Metal oxides are widely studied for oxidation reaction (Fang et al., 2013; Hao et al., 2013; Zhang et al., 2013; Li et al., 2014; Qadir et al., 2014; Gui et al., 2015; Imanaka et al., 2015; Wang et al., 2016; Shiraishi et al., 2017). The nature of the metal oxides as support or as active species influenced the catalytic performance of the catalysts significantly (Unnarkat et al., 2016; Ribeiro de Sousa Martins et al., 2017; Yang et al., 2017; Feliciano Miranda et al., 2018). Acharyya et al. (2015) synthesized Cr_2_O_3_ supported Cu nanoclusters with hydrothermal method which converted cyclohexane to cyclohexanone with high yield, but failed to produce any AA. Whereas, WO_3_ supported Cu converted cyclohexane with 88% conversion and 75% selectivity to AA (Table 2, #1) (Acharyya et al., 2015). Most probably, the activation energy was lowered in the case of Cu-WO3 catalysts due to the flexibility property of the Cu-framework. The impregnated CuO/WO_3_ catalyst was inactive for cyclohexane oxidation to AA. It seems that the synergistic interaction between the Cu and W species is the main reason of the oxidation activity. Recycled Cu-WO_3_ catalysts have no metal leaching in at least four consecutive runs without any decreasing of catalytic performance. Comparing to Cu, Au has the same outermost electronic configurations but far higher activity in oxidation reactions. Liu et al. (2016) coated Au on the wall of the stainless steel microcapillary. A conversion of 2.1% and selectivity of 18.9% to AA were obtained for cyclohexane oxidation in 4 min (Table 2, #2). The stability of the catalyst was not reported in this work. Alshammari et al. (2015, 2016) incorporated Au, Pd, and Ag on TiO_2_ using sol-gel methods. Bimetallic catalysts AuPd/TiO_2_ showed higher selectivity compared to monometallic Pd/TiO_2_ toward AA (Table 2, #3) due to smaller particle size as observed by TEM images. Au as a second metal is important for enhancing the AA selectivity due to the synergistic effects between Au and Pd metals. The bimetallic catalyst was observed deactivated after consecutive runs due to the formation of Pd^δ−^ species with lower binding energy, metal leaching and coke formation (Alshammari et al., 2016). Chen et al. (2017) confined Au nanoparticles in hybrid shells of organic linker-assisted silica nanospheres (GOS) using amino function groups for anchoring Au precursor. TEM images showed that GOS has uniformed nanospheres with 120–150 nm diameter. The AuNPs (<2 nm) were highly dispersed on the shells of silica. FTIR and Raman results indicated that the incorporation of AuNPs didn't alter the structure of GOS. The obtained catalyst oxidized cyclohexane with 45% selectivity to AA under solvent-free conditions using O_2_ as oxidant (TOF = 59,307 h^−1^, Table 2, #4). It seems that the AuNPs confined in silica shell is more active than that in the inner cores. Besides, C-H bonds in silica shell improved the hydrophobicity and the adsorption of cyclohexane.

**Table 2:** Cyclohexane, cyclohexanol, and cyclohexanone oxidation to AA.

**#**	**Catalyst**	**Reaction conditions**	**Conversion, selectivity**
1	Cu-WO_3_	Cyclohexane, 70°C, 12 h, H_2_O_2_	X = 75%, S = 88%, TON = 119
2	Au-Al_2_O_3_	Cyclohexane, 180°C, 0.25 h, 3MPa, O_2_	X = 2.1%, S = 18.9%
3	Au/TiO_2_	Cyclohexane, 150°C, 4 h, TBHP, 1MPa, O_2_	X = 25%, S = 26%, TON=237
4	AuNPs(GOS)	Cyclohexane, 150°C, 3 h, TBHP	X = 34%, S = 45.1%, TON = 59307
5	Mn-HTS	Cyclohexane, 140°C, 6 h, 1MPa, O_2_	X = 8.6%, S = 57.7%, TON = 324
6	W/HTS	Cyclohexane, 90°C, 14 h, H_2_O_2_	X = 31.4%, S = 78.5%, TON = 31
7	Fe@CNT-100	Cyclohexane, 125°C, 8 h, 1.5MPa O_2_	X = 39.7%, S = 49.7%, TON = 299
8	M-PW_12_O_40_	Cyclohexene, 100°C, 72 h, H_2_O_2_	X = 75%, Y = 61%
9	Al_2_O_3_@Fe_2_O_3_	Cyclohexanone, 80°C, 24 h, H_2_O_2_	TON = 71
10	Mn-HTS	Cyclohexanone, 90°C, 9 h, 0.6Mpa, O_2_	X = 68%, S = 93%, TON = 713
11	Mn- HMTS	Cyclohexanone, 90°C, 8 h, 0.6Mpa, O_2_	X = 64%, S = 94%, TON = 887
12	TS-1	Cyclohexanone, 80°C, 8 h, H_2_O_2_	X = 53%, S = 33%, TON = 34
13	FePO-1-2	Cyclohexanone, 75°C, 10 h, 0.1Mpa, O_2_	X = 72%, S = 96%, TON = 42
14	TIPO-1	Cyclohexanone, 80°C, 8 h, H_2_O_2_	X = 92%, S = 66%, TON = 49
15	MnAPO-5	Cyclohexanone, 85°C,72 h, TBHP	X = 100%, S = 100%, TON = 566
16	NH_4_SnPMo_12_O_40_	Cyclohexanone, 90°C, 20 h, H_2_O_2_	X = 100%, S = 56
17	HNi_1.5_PMo_12_	Cyclohexanone, 90°C, 20 h, H_2_O_2_	Y = 31%
18	CoPMo_12_O_40_	Cyclohexanone, 90°C, 20 h, H_2_O_2_	Y = 75.5%
19	H_3+x_PMo_12−x_V_x_O_40_	Cyclohexanone, 70°C, 12 h, 0.41MPa, air	X = 16%, S = 42%,
20	K_6_P_2_Mo_6_W_12_O_62_	Cyclohexanol, 90°C, 20 h, H_2_O_2_	Y = 59%

Hollow structure silicates (HTS) with large intraparticle voids were more active than TS-1 catalyst for cyclohexane oxidation reaction as reported (Shi et al., 2011). This special structure can aggravate the movement of products and reactants in and out of the channels. Zou et al. (2015b) evaluated various of HTS catalysts and found Mn-HTS gave the highest selectivity toward AA due to the nature of Mn metal (Table 2, #5). The stability of Mn-HTS catalysts maintained in four runs. The stability was confirmed by comparing the FT-IR and UV-Vis spectra of the fresh and recycled catalysts. This reaction proceeded via radical intermediates with Ti(IV)-O• or Ti(IV)-OO• species as active centers and Mn^3+^ as promoters. W based HTS bifunctional catalysts showed higher activity compared to H_2_WO_4_/TS-1 for the oxidation of cyclohexane (Table 2, #6) due to higher accessibility of Ti species, large intraparticle voids and the bifunctional catalytic sites (Dai et al., 2016).

Carbon nanotubes (CNTs) have been widely used as catalysts support because they are insoluble in the most solvents (Coleman et al., 2006; Moniruzzaman and Winey, 2006; Tangestaninejad et al., 2008, 2009; Moghadam et al., 2010a,b). On the other hand, CNT can create confined spaces for metals to prevent the aggregation and to act as a template for metal seed growth (Moghadam et al., 2010a). Yang et al. (2016) prepared Fe-, Ni-, and FeNi- based CNT catalysts with controllable wall thickness and evaluated for cyclohexane oxidation. They found that Fe@CNT showed highest catalytic performance (Table 2, #7) ascribing to the thin walls of CNTs and confined electron-donating metals, which will help the electron transfer on the CNTs surfaces (Yang et al., 2016). Besides, the Fe filling can enhance the electronic property of the graphene sheets. Ni@CNT has lower activity due to the weaker interaction with carbon.

**Figure 3:**
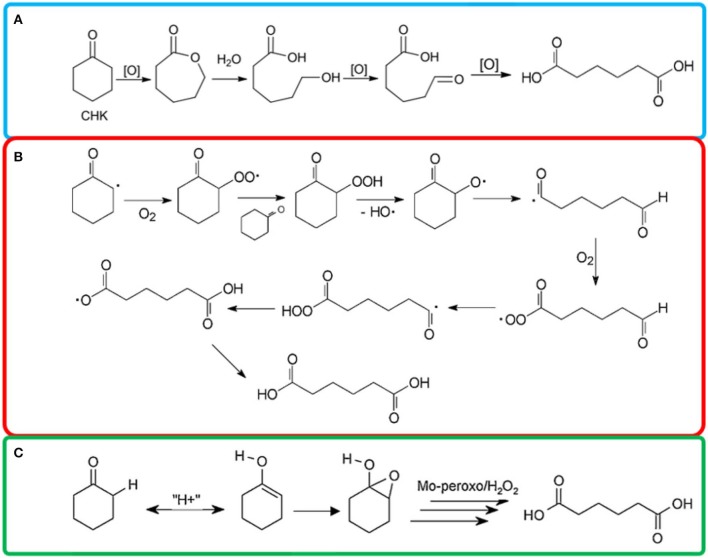
Proposed **(A)** Baeyer-Villiger oxidation type of mechanism, (Pisk et al., 2019) **(B)** radical chain autoxidation mechanism, (Cavani et al., 2011) **(C)** redox mechanism (Amitouche et al., 2018) of cyclohexanone oxidation to AA.

POMs were reported highly active for oxidation reaction to synthesize AA with a TON values as high as 29,550 (Luo et al., 2018) The exceptional performance of POMs was possibly due to the fact that POMs played the roles of co-catalysts, active metal sites stabilizer and electronic structure regulator in the oxidation process (Banerjee et al., 2012; Tahar et al., 2015). Keggin type POMs, were the most studied type POMs for liquid phase oxidation, due to their high resistance to oxygen donors and strong oxidizing power (Wang et al., 2015; He et al., 2016; Yu et al., 2017). Pisk et al. (2019) reported Merrifield resins supported Mo- or W- based Keggin POMs as catalysts to oxidize cyclohexene to AA with 46 and 61% yield (Table 2, #8). They found the W based POMs are more active than Mo based catalysts. They proposed Baeyer-Villiger oxidation type of mechanism for this process (Figure 3A).

### Cyclohexanone Oxidation to AA

Patra et al. (2013) developed a method to encapsulate γ-Al_2_O_3_ nanoparticles by a thin shell of α-Fe_2_O_3_. The resulting material showed high surface area and meso-porosity due to self-aggregation of tiny spherical nanocrystals as confirming by SEM images. These catalysts displayed low TON for the oxidation of cyclohexanone to AA in water (Table 2, #9), because only the surface Fe center took part in the reaction. A single layer of Fe center on the surface of the core was believed to enhance the performance of the catalysts.

Mn-HTS catalysts displayed the good performance with 68% cyclohexanone conversion, 93% AA selectivity (Table 2, #10) (Zou et al., 2015a). Mn-HTS, with high oxidation states, had less BrØnsted acid sites than Lewis acid sites which favored the formation of enolate from the keto-form of cyclohexanone (Zou et al., 2015a). The recycled catalysts were shown to maintain the same Mn and Ti content in about 15 cycles of reuse. They also noticed that the use of acetic acid as the co-solvent can form CH_3_COOOH as oxidizing species and thus improve the reaction rate and AA selectivity. Some other groups also noticed the same phenomenon and claimed that the reaction proceed via a radical-chain autoxidation mechanism, rather than a redox mechanism in the presence of acetic acid (Shimizu et al., 2003; Cavani et al., 2011). On the other hand, acetic acid can stabilize the H_2_O_2_ and prevent the decomposition (Chavan et al., 2002; Shimizu et al., 2003). Gao's group prepared Mn-HMTS catalyst by a one-step hydrothermal approach with tunable textural properties (Table 2, #11, TON = 887) (Gao et al., 2019). They noticed that the textural and physicochemical properties of Mn-HMTS can be easily tuned by modifying the amounts of the template agent. Free-radical mechanism was proposed, since Mn species acted as a promoter for both radical intermediates and enol formation from cyclohexanone (Gao et al., 2019). Xia's group also studied TS-1 catalysts for cyclohexanone oxidation reaction by combining density function theory (DFT) calculation with experimental studies (Table 2, #12) (Xia et al., 2015). DFT calculations indicated that H_2_O_2_ molecule was absorbed and activated at the tetrahedral Ti sites.

Phosphonate based metal catalysts have immense potential to be used as ecofriendly catalysts due to the high durability and thermal stability (Zhao et al., 2006; Deng et al., 2011; Dutta et al., 2012; Mahdavi and Hasheminasab, 2015; Xiao et al., 2016; Rezaei et al., 2017). Bhanja et al. (2016) synthesized an organic-inorganic hybrid iron phosphonate materials (FePO-1-2) via a hydrothermal synthesis route. This material displayed high activity for the cyclohexanone oxidation due to the high surface acidity as well as the framework redox Fe^II/III^ sites (Table 2, #13). They also observed that water show more remarkable promotion effect and good AA selectivity due to the higher polarity than other solvents (Bhanja et al., 2016). There was only very slight decrease of the AA yield after six consecutive reactions. The XRD results suggested there was only minor decrease in the crystallinity and BET surface area. There was no detectable Fe leaching from the catalyst. Later, the same group developed a oxyfluorinated titanium phosphate material (TIPO-1, Table 2, #14) (Bhanja et al., 2018). This material showed a 92% cyclohexanone conversion and 66% selectivity to AA. A Mn incorporating aluminophosphate material (MnAPO-5) was synthesized by Chatterjee's group (Chatterjee et al., 2018). A complete conversion of cyclohexanone and AA selectivity were obtained (Table 2, #15). The detected ε-caprolactone as intermediate by ^1^H NMR. They proposed a reaction pathway that ε-caprolactone formed by Baeyer-Villiger oxidation and then the ring undergoes oxidative C-C bound cleavage to give AA. No leaching of Mn was detected at the end of each run.

Mouheb et al. (2018) synthesized Keggin-type POMs (Table 2, #16) and revealed that the active species for cyclohexanone oxidation might be the peroxo-polyoxometalates (Mouheb et al., 2018). On the other hand, more unidentified products formed when cyclohexanol was used as substrate. This catalyst can be reused at least 3 times without regeneration. Amitouche et al. (2018) synthesized Keggin heteropolyacid catalyst. They disclosed the pathways to different H_3_PMo_12_O_40_ reduced state and the transformation into peroxomolybdate complexes (Amitouche et al., 2018). As shown in Figure 3B, the H-abstraction at the carbon next to the oxygen in cyclohexanone can be promoted in the presence of active species, and the production of radical reacted with oxygen and formed cyclohexyl hydroperoxide (Zou et al., 2015a). The ketonyl radical underwent ring opening via C–C cleavage and formed OHC–(CH_2_)_4_-C(O) radical species (Amitouche et al., 2018). The last step was oxidation that lead to the formation of AA. H_3−2x_Ni_x_PMo_12_O_40_ catalysts showed AA yield of 31% (Table 2, #17) (Tahar et al., 2015). The results showed that the AA yield was sensitive to the chemical composition and the x value. H_3−2x_Co_x_PMo_12_O_40_ (x: 0–1.5) catalysts were prepared using the cationic exchange method (Benadji et al., 2013). The cobalt salts were more effective than parent acid to oxidize cyclohexanone (*X* = 76%) and cyclohexanol (*X* = 53%) because the Co-based POMs acted as acidifying and oxidizing agent (Table 2, #18). H_3+x_PMo_12−x_V_x_O_40_ catalyzed cyclohexanone oxidation via a redox mechanism and the reoxidation of the reduced POM was the rate-limiting step (Table 2, #19) (Cavani et al., 2011). However, when an acetic acid was used as additive, a radical chain autoxidation mechanism prevailed. The metal composition of the POMs affected the relative importance of the two mechanism (Figures 3B,C). The radical chain autoxidation mechanism was more selective to AA than the redox mechanism, because in the radical chain autoxidation mechanism there was no intermediate of partially oxidized products (lighter acids and CO_2_) formed (Cavani et al., 2011).

Anderson and Dawson type POMs were also reported active for oxidation reactions. Luo et al. (2018) synthesized a POMs nanoclusters with butterfly-shaped β isomer. This catalyst displayed good activity (TON: 29,550) toward AA in solvent free condition. When cyclohexanol was used as substrate, the AA yield was lower than the cyclohexanone. The recycle ability study indicated that there was an appreciable loss of AA yield after three runs. Dawson-type POMs (P_2_M_18_) have potential to have oxidation properties since they have more elements with a high oxidation state than that of the Keggin anion (Moudjahed et al., 2016). Moudjahed et al. (2016) prepared Dawson-type POMs which showed an AA yield of 69% in the KA oil oxidation reaction (Table 2, #20). ^31^P NMR spectroscopy of used POMs confirmed the formation of “peroxo-POMox” as active intermediate species.

## Conclusion

Based on the critical review, biomass-based AA provides important and alternative routes for future development of nylon industry. At present, selective oxidation of sugars and derivatives to relevant aldaric acids is the key challenge in this area. Future work should be focused on finding more effective and inexpensive materials to achieve this chemistry. The progress and potential significance of nanostructured solid catalysts and POMs catalysts for oxidation of cyclohexane, cyclohexene, cyclohexanol and cyclohexanone to produce AA with green oxidants have been critically revised in this paper. This work summarized and discussed catalysts synthesis and structural characterization, the oxidation reaction mechanism, as well as catalyst durability. The POMs with dual redox and acidity properties display high catalytic activity and selectivity for cyclohexane/cyclohexene/cyclohexanone/cyclohexanol oxidation. Important accomplishments in this research area could be further achieved by the efficient catalyst design, and a deep understanding of both redox and radical based oxidation mechanisms.

Fundamental understanding of catalysts deactivation and oxidant utilization efficiency improvement should be the focusing efforts in the future study. The economic and environment analysis of the new green processes are needed to systematically study to see if the green processes has the potential to replace in the current industrial process. This work provides guidance for further investigation on metal nano catalysts for the efficient, green, safe, sustainable, ecofriendly and economical route of AA production and oxidation processes for many other value-added fine chemicals production.

## Author Contributions

WY drafted the manuscript. XJ conceived the concept of the review. GZ, JW, and ML conducted literature survey. YS, ZZ, WZ, and SZ organized figures and revised the manuscript. XX and JS provided comments.

### Conflict of Interest

XX was employed by the company Huabei Oil Field Company at PetroChina. The remaining authors declare that the research was conducted in the absence of any commercial or financial relationships that could be construed as a potential conflict of interest.
